# Efficacy and safety of tyrosine kinase inhibitors in advanced hepatocellular carcinoma patients with Child-Pugh A and B cirrhosis: a meta-analysis

**DOI:** 10.3389/fphar.2026.1690890

**Published:** 2026-03-11

**Authors:** Xionglin Liu, Jindu Li, MinJun Li, Bangde Xiang

**Affiliations:** 1 Department of Hepatobiliary Surgery, Guangxi Medical University Cancer Hospital, Nanning, Guangxi, China; 2 Key Laboratory of Early Prevention and Treatment for Regional High Frequency Tumor, Ministry of Education, Nanning, China

**Keywords:** Child–Pugh classification, hepatocellular carcinoma, meta-analysis, treatment-related adverse events, tyrosine kinase inhibitors

## Abstract

**Background:**

Hepatocellular carcinoma (HCC) remains a leading cause of cancer mortality. Tyrosine kinase inhibitors (TKIs) are widely used in advanced HCC, yet outcomes may differ by hepatic reserve. We compared the efficacy and safety of guideline-recommended TKIs between patients with Child–Pugh A (CP-A) and Child–Pugh B (CP-B) cirrhosis.

**Methods:**

We conducted a PRISMA-guided systematic review and meta-analysis (searches through 15 October 2024). Eligible studies evaluated TKI monotherapy and reported outcomes separately for CP-A and CP-B. Primary endpoints were overall response rate (ORR), disease control rate (DCR), and grade ≥3 treatment-related adverse events (trAEs). Pooled risk ratios (RRs) with 95% CIs were estimated using prespecified random-effects models; small-study effects were explored with funnel plots (and Egger’s test when k ≥ 10).

**Results:**

Twenty-four studies met inclusion. Pooled analyses showed no significant differences between CP-B and CP-A for ORR (k ≈ 15; RR 1.02, 95% CI 0.83–1.27; I^2^ 42.8%) and DCR (k ≈ 10; RR 0.87, 95% CI 0.58–1.29; I^2^ 83.1%). For grade ≥3 trAEs (k ≈ 9), CP-A had a lower risk than CP-B (CP-A vs. CP-B RR 0.79, 95% CI 0.63–1.00; I^2^ 61.7%), indicating a borderline increase in severe toxicity among CP-B. Funnel plots showed no clear asymmetry; Egger’s test for ORR was negative (p = 0.94), and tests were not performed for DCR and trAEs due to k < 10.

**Conclusion:**

In advanced HCC treated with TKI monotherapy, radiologic efficacy (ORR, DCR) appears broadly comparable between CP-B and CP-A, whereas CP-B may experience higher rates of grade ≥3 toxicities. TKIs remain a reasonable option for selected CP-B patients, provided dosing is individualized and adverse events are closely monitored. Prospective, stratified studies are needed to refine patient selection and dose management in CP-B.

## 1 Introduction

Hepatocellular carcinoma (HCC) is the sixth most common cancer worldwide and the fourth leading cause of cancer-related mortality (Brown et al., 2023). Advanced HCC presents a significant therapeutic challenge due to its poor prognosis and limited treatment options. Tyrosine kinase inhibitors (TKIs) such as sorafenib, lenvatinib, regorafenib, and cabozantinib have been widely used as systemic therapies for advanced HCC (NCCN, 2024). These TKIs function by inhibiting multiple signaling pathways involved in tumor proliferation and angiogenesis, thereby slowing disease progression (Fallahi et al., 2022).

In the landscape of systemic treatments for advanced HCC, TKIs are second only to immune checkpoint inhibitors (ICIs) in terms of efficacy (Sun et al., 2023; Jin et al., 2024). While ICIs have shown promising results and have been the focus of numerous studies evaluating their safety and efficacy, TKIs remain a cornerstone of HCC management, particularly as salvage therapies for patients who are not candidates for or have progressed on ICIs (NCCN, 2024; Giovanis et al., 2018). Recent studies have highlighted the role of macrophage reprogramming in enhancing the efficacy of ICIs and TKIs in HCC, underscoring the importance of targeting the tumor microenvironment (Wang et al., 2024). Additionally, emerging therapies, such as those involving tumor-derived exosomes, have shown promise in inhibiting metastasis and overcoming resistance to conventional treatments, representing a new frontier in HCC management (Jia et al., 2024).

Despite their widespread use, previous studies have primarily focused on comparing the efficacy and safety of single-agent TKIs, such as sorafenib, between patients with different degrees of liver function (Mcnamara et al., 2018). These studies often evaluated sorafenib alone and did not integrate data on other guideline-recommended TKIs, leaving a gap in understanding the collective efficacy and safety profiles of multiple TKIs in patients with varying liver function statuses.

Given that liver function is a pivotal factor influencing both the pharmacokinetics of TKIs and the patient’s ability to tolerate therapy, it is essential to understand how liver dysfunction impacts treatment outcomes. Patients with Child-Pugh B (CPB) cirrhosis represent a particularly vulnerable group, often exhibiting altered drug metabolism and increased risk of adverse events (Turnes et al., 2015; Griffiths et al., 2022; Stefanini et al., 2024). Previous studies suggest that TKIs may be more effective in advanced HCC patients with Child-Pugh B classification (Ji et al., 2014; Labeur et al., 2020). However, there has been a lack of comprehensive analyses integrating data on multiple commonly used TKIs to compare their efficacy and safety between Child-Pugh A (CPA) and CPB patients in advanced HCC.

Therefore, this meta-analysis aims to fill this gap by systematically evaluating and integrating the efficacy and safety data of multiple guideline-recommended TKIs—including sorafenib, lenvatinib, regorafenib, and cabozantinib—in advanced HCC patients stratified by Child-Pugh classification. By doing so, we seek to provide clinicians with a more complete evidence-based guidance on the use of TKIs in patients with compromised liver function. This work represents a novel contribution to the existing literature, as it is the first to comprehensively compare the therapeutic outcomes of multiple TKIs between CPA and CPB patients in advanced HCC. Understanding these differences is crucial for optimizing treatment strategies, improving patient outcomes, and minimizing adverse events in this vulnerable patient population.

## 2 Methods

### 2.1 Protocol and registration

This systematic review and meta-analysis were conducted following the Preferred Reporting Items for Systematic Reviews and Meta-Analyses (PRISMA) guidelines. The study protocol was registered on the International Platform of Registered Systematic Review and Meta-analysis Protocols (INPLASY) under the registration number INPLASY2024100130.

### 2.2 Inclusion and exclusion criteria

#### 2.2.1 Inclusion criteria

Studies were included if they met the following criteria: they were randomized controlled trials, cohort studies, or case-control studies involving patients with advanced hepatocellular carcinoma (HCC) who received tyrosine kinase inhibitors (TKIs) as monotherapy. Participants were adult patients aged 18 years or older, diagnosed with advanced HCC and classified as Child-Pugh class A or B liver cirrhosis. The studies provided comparative data between patient groups with Child-Pugh class A and B. Additionally, they reported at least one of the following outcomes: overall response rate (ORR), disease control rate (DCR), or incidence of grade 3 or higher treatment-related adverse events (TrAEs).

#### 2.2.2 Exclusion criteria

Studies were excluded if they did not focus on primary liver cancer or involved other types of cancers. Those lacking clear information on Child-Pugh grading were also excluded. Furthermore, studies where TKIs were used in combination with other treatments, such as immunotherapy or chemotherapy, were not included.

### 2.3 Information sources

We conducted a comprehensive search of electronic databases up to 15 October 2024, including PubMed, Web of Science, and the Cochrane Library. In addition, we manually searched the reference lists of relevant articles and reviews to identify other eligible studies. No restrictions were placed on publication status.

### 2.4 Search strategy

A search strategy was developed using Medical Subject Headings (MeSH) terms and free-text keywords related to tyrosine kinase inhibitors, hepatocellular carcinoma, Child-Pugh classification, and advanced disease stages. The detailed search terms and strategies are provided in the protocol INPLASY2024100130.

### 2.5 Selection process

Two independent reviewers (Reviewer A and Reviewer B) initially screened the titles and abstracts of all retrieved records to identify potentially eligible studies. For studies deemed relevant, full texts were obtained and independently assessed according to the predefined inclusion and exclusion criteria. Disagreements were resolved through discussion or consultation with a third reviewer (Reviewer C). The selection process was documented using a PRISMA flow diagram (Supplementary Figure S1), as shown in the Supplementary Material. No automation tools were used in the selection process. The quality of the studies was assessed using the Newcastle-Ottawa Scale (NOS), with detailed scores provided in the supplement (Supplementary Table S1).

### 2.6 Data collection process

Two reviewers (Reviewer A and Reviewer B) independently extracted data using a standardized data extraction form. Extracted data were cross-checked to ensure accuracy and completeness. Any discrepancies were resolved through discussion or by consulting a third reviewer (Reviewer C). No automation tools were used for data collection.

### 2.7 Data items

From each included study, we extracted information on study characteristics (authors, publication year, country, study design, and sample size), participant characteristics (age, gender, and Child-Pugh classification [A or B]), and intervention details (specific TKI drugs used and dosages). Outcome measures were also extracted, including efficacy outcomes such as ORR and DCR assessed according to RECIST or mRECIST criteria, and safety outcomes such as the incidence of grade 3 or higher TrAEs reported according to the version of the Common Terminology Criteria for Adverse Events (CTCAE) used. For missing or unclear information, assumptions were made based on the best available data in the studies when necessary.

### 2.8 Effect measures

We calculated risk ratios (RRs) with 95% confidence intervals (CIs) for dichotomous outcomes (objective response rate [ORR], disease control rate [DCR], and grade ≥3 treatment-related adverse events [trAEs]). A continuity correction of 0.5 was applied when necessary.

### 2.9 Publication bias assessment

To avoid over-testing, we assessed small-study effects using visual inspection of funnel plots and Egger’s regression test only when ≥10 studies contributed to an outcome. We did not run additional overlapping tests. Results were interpreted cautiously because these procedures evaluate small-study effects (i.e., potential publication bias) rather than within-study risk of bias.

### 2.10 Synthesis methods

Meta-analyses were conducted in R (meta). Because between-study heterogeneity was anticipated a priori, random-effects models were prespecified; common-effect (fixed-effect) estimates are shown for reference only, and model choice was not based on observed heterogeneity. Dichotomous outcomes (ORR, DCR, and grade ≥3 treatment-related AEs) were pooled as risk ratios (RRs) on the log scale using inverse-variance weighting, with between-study variance τ^2^ estimated by REML and Hartung–Knapp adjustments applied to random-effects confidence intervals. Heterogeneity was summarized with I^2^ (and Q), and 95% prediction intervals were reported for random-effects summaries. For sparse data, a small continuity correction (0.5) was used when required. Robustness was examined through prespecified sensitivity analyses (e.g., restricting to higher-quality studies and using alternative τ^2^ estimators such as Paule–Mandel.).

### 2.11 Reporting bias assessment

Reporting bias was assessed using funnel plots, which are displayed in Figures 4–6. The results of Egger’s tests are presented in the figure legends.

### 2.12 Certainty assessment

Due to heterogeneity in study designs and outcomes, as well as the limited number of available studies, a formal certainty assessment of the evidence (e.g., using the GRADE approach) was not performed. This limitation is acknowledged in the Discussion section.

### 2.13 Ethical considerations

This study was based on publicly available published data; therefore, ethical approval and informed consent were not required.

### 2.14 Supplementary materials

Detailed search strategies, the PRISMA flow diagram, and the collected data are included in the Supplementary Material.

## 3 Results

### 3.1 Study selection

A total of 936 relevant articles were identified, including 782 from database searches and 154 from other sources (such as manual searches of reference lists). After screening titles and abstracts, 868 articles that did not meet the inclusion criteria were excluded. The remaining 68 articles underwent full-text evaluation, and 44 studies were further excluded for reasons such as lacking data on Child-Pugh classification, not reporting the required outcomes, involving combination therapies, or focusing on non-HCC patients. Ultimately, 24 studies were included in both qualitative and quantitative analyses.

### 3.2 Study characteristics

The 24 included studies were published between 2009 and 2023 and comprised randomized controlled trials, prospective cohort studies, and retrospective cohort studies from various countries and regions. All studies evaluated the efficacy and safety of tyrosine kinase inhibitors (TKIs) monotherapy in patients with advanced hepatocellular carcinoma (HCC), comparing outcomes between Child-Pugh A and Child-Pugh B patient groups. Detailed characteristics of the included studies are presented in Tables 1, 2.

**TABLE 1:** Incidence of Grade 3–4 trAEs in HCC Patients by Child-Pugh Class Treated with TKIs.

Author	Year	Follow-up time	Agent	Events (n)/Patients (n)	
				Child-pugh A	Child-pugh B
Lencioni et al. (2012)	2013	1 year	sorafenib	228/957	80/367
Ye et al. (2016)	2018	1.5–2 years	sorafenib	9/246	2/48
Turnes et al. (2015)	2015	2 years	sorafenib	24/96	9/35
Suzuki et al. (2018)	2018	1–2 years	sorafenib	31/40	11/12
Komiyama et al. (2020)	2020	1.5 years	regorafenib	5/13	2/8
Kim et al. (2020)	2020	2 years	regorafenib	61/440	16/59
Cheon et al. (2020)	2020	1 year	lenvatinib	22/74	5/18
Marrero et al. (2016)	2015	>1 year	sorafenib	638/1968	210/666
da Fonseca et al. (2015)	2015	2 years	sorafenib	20/100	3/20
De Guevara et al. (2020)	2020	2 years	Sorafenib	2/33	8/41

HCC, hepatocellular carcinoma; TKI, tyrosine kinase inhibitor; TrAEs, Treatment-Related Adverse Events.

**TABLE 2:** ORR and DCR in HCC patients by child-pugh class treated with TKIs.

Author	Year	Follow-up time	Agent	ORR		DCR	
				Events (n)/Patients (n)		Events (n) patients (n)	
				CP-A	CP-B	CP-A	CP-B
Suzuki et al. (2018)	2018	1–2 years	sorafenib	4/40	0/12	23/40	8/12
Camargo-Pinheiro-Alves et al. (2019)	2019	2 years	Sorafenib	44/108	25/65	​	​
Singal et al. (2023)	2023	1 year	lenvatinib	33/51	41/81	​	​
Pinter et al. (2009)	2009	1 year	Sorafenib	0/26	1/23	5/26	8/23
Persano et al. (2023)	2022	3 years	lenvatinib	453/1166	53/146	​	​
Woo et al. (2012)	2012	4 months	Sorafenib	18/64	4/16	11/64	3/16
Chiu et al. (2012)	2012	2 years	Sorafenib	4/108	2/37	23/108	16/64
Køstner et al. (2013)	2012	2 years	Sorafenib	5/43	2/29	​	​
Huynh et al. (2022)	2022	2 years	Lenvatinib/Sorafenib	233/840	21/107	582/840	73/107
Cheon et al. (2020)	2020	1 year	lenvatinib	12/74	1/18	62/74	11/18
Abou-Alfa et al. (2011)	2010	2–5 years	Sorafenib	3/98	0/38	68/98	17/98
Kim et al. (2011)	2011	3 years	Sorafenib	2/199	2/68	106/199	19/68
Maruta et al. (2020)	2020	0.5–1.5 years	Lenvatinib	52/130	10/20	91/130	13/20
Zugazagoitia et al. (2013)	2013	2 years	Sorafenib	2/30	0/18	14/30	8/18
Kim et al. (2013)	2013	2 years	sorafenib	4/245	1/80	99/245	16/80
Ozenne et al. (2010)	2010	1 year	sorafenib	3/33	1/17	21/33	6/17

HCC, hepatocellular carcinoma; TKI, tyrosine kinase inhibitor; ORR, objective response rate; DCR, disease control rate; CP, Child-Pugh; TrAEs, Treatment-Related Adverse Events.

### 3.3 Main outcomes

#### 3.3.1 Incidence of grade ≥3 treatment-related adverse events (trAEs)

**FIGURE 1:**
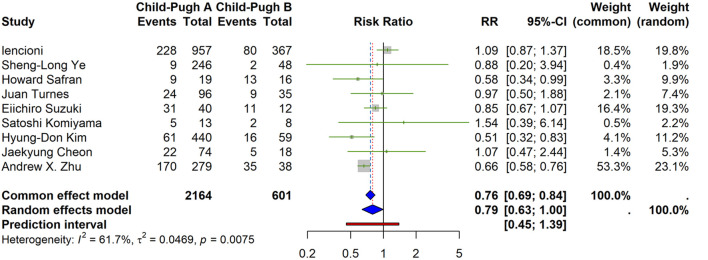
Incidence of grade ≥3 treatment-related adverse events (trAEs) by Child–Pugh class (forest plot). Random-effects summary RR = 0.79 (95% CI 0.63–1.00), indicating a lower risk in CP-A vs. CP-B with borderline significance. Heterogeneity: I^2^ = 61.7%, τ^2^ = 0.0469, Q p = 0.0075. Prediction interval: 0.45–1.39. Fixed-effect estimate in gray is presented for reference only.

Under the prespecified random-effects model, the pooled risk ratio (RR) was 0.79 (95% CI 0.63–1.00), indicating a lower risk of grade ≥3 trAEs in CP-A than CP-B, with borderline statistical significance. Between-study heterogeneity was moderate (I^2^ = 61.7%, Q *p* = 0.0075; τ^2^ = 0.0469). The 95% prediction interval (PI) was 0.45–1.39, suggesting the true effect could vary from benefit to no material difference across settings (Figure 1).

#### 3.3.2 Overall response rate (ORR)

**FIGURE 2:**
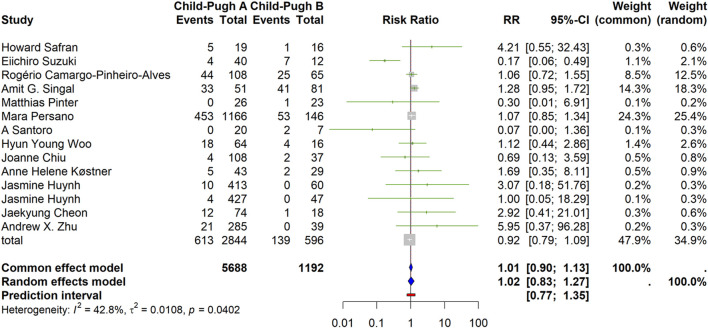
Overall response rate (ORR) by Child–Pugh class (forest plot). Random-effects summary RR = 1.02 (95% CI 0.83–1.27), showing no significant difference between CP-A and CP-B. Heterogeneity: I^2^ = 42.8%, τ^2^ = 0.0108, Q p = 0.0402. Prediction interval: 0.77–1.35.

The random-effects model showed no significant difference between CP-A and CP-B (RR = 1.02, 95% CI 0.83–1.27). Heterogeneity was moderate (I^2^ = 42.8%, Q *p* = 0.0402; τ^2^ = 0.0108). The PI was 0.77–1.35, consistent with no material difference (Figure 2).

#### 3.3.3 Disease control rate (DCR)

**FIGURE 3:**
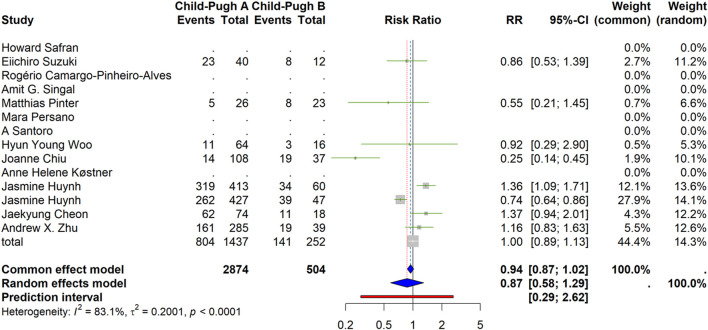
Disease control rate (DCR) by Child–Pugh class (forest plot). Random-effects summary RR = 0.87 (95% CI 0.58–1.29), indicating no significant difference. Heterogeneity: I^2^ = 83.1%, τ^2^ = 0.2001, Q p < 0.0001. Prediction interval: 0.29–2.62.

For DCR, the random-effects summary was RR = 0.87 (95% CI 0.58–1.29), indicating no significant difference between CP-A and CP-B. Heterogeneity was substantial (I^2^ = 83.1%, Q *p* < 0.0001; τ^2^ = 0.2001). The PI was 0.29–2.62, reflecting considerable between-study variability (Figure 3).

### 3.4 Heterogeneity and subgroup analyses

Overall, heterogeneity was moderate for trAEs and ORR and substantial for DCR. Exploratory subgroup analyses by response evaluation criteria (RECIST vs. mRECIST) and by TKI agent/class did not materially reduce heterogeneity. Together with the wide prediction intervals, this suggests that between-study variability likely reflects differences in population mix and baseline liver function, line of therapy, starting-dose/dose-modification strategies, and study design/setting, rather than the stratifiers examined.

### 3.5 Sensitivity analyses

Pre-specified sensitivity analyses—using the Paule–Mandel estimator for τ^2^, restricting to higher-quality studies (e.g., NOS ≥7), and excluding overlapping cohorts/reports—yielded results directionally consistent with the primary analyses. The trAEs outcome continued to show a lower risk in CP-A, with confidence intervals generally close to 1; ORR and DCR remained non-significant. Heterogeneity indices varied across subsets and methods, but overall interpretation was unchanged.

### 3.6 Reporting bias assessment

**FIGURE 4:**
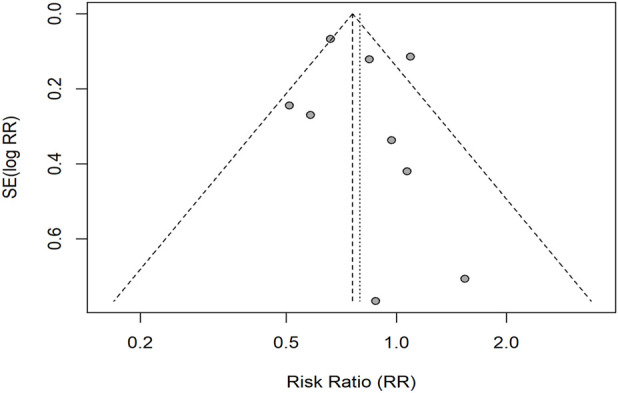
Funnel plot for grade ≥3 trAEs (RR scale). Points depict individual studies; dashed diagonals indicate pseudo 95% confidence limits; the vertical dashed line marks the random-effects pooled RR. Egger’s test was not conducted (k < 10); visual inspection showed no clear asymmetry.

**FIGURE 5:**
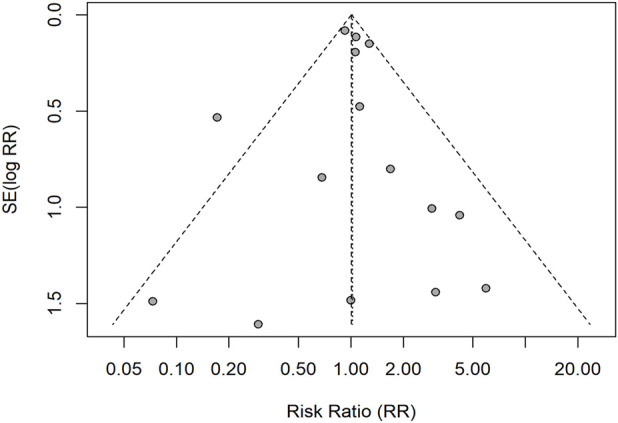
Funnel plot for ORR (RR scale). Dashed diagonals show pseudo 95% limits; the vertical dashed line is the random-effects pooled RR. Egger’s linear regression: intercept 0.03 (SE 0.47), *t* = 0.07, *p* = 0.94 (k = 15), indicating no evidence of small-study effects.

**FIGURE 6:**
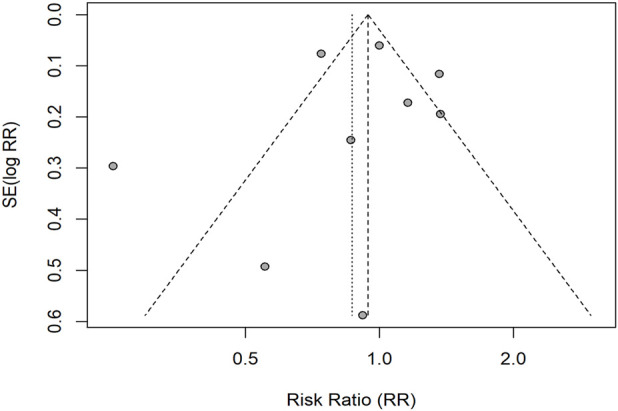
Funnel plot for DCR (RR scale). As in Figure 6. After excluding records with missing data, k = 9 remained; per the a priori plan (tests only when k ≥ 10), Egger’s test was not conducted. Visual inspection showed no obvious asymmetry.

Funnel plots were examined for each outcome (Figures 4–6). For ORR (k = 15), Egger’s linear regression provided no evidence of small-study effects (intercept 0.03, SE 0.47; *t* = 0.07; *p* = 0.94). For DCR, after excluding records with missing data, nine studies remained; per our a priori plan (tests only when k ≥ 10), Egger’s test was not conducted and the funnel plot was inspected visually. For grade ≥3 trAEs (k = 9), Egger’s test was likewise not conducted (<10 studies). Overall, no clear funnel-plot asymmetry was observed. These procedures evaluate small-study effects (potential publication bias) rather than within-study risk of bias.

## 4 Summary

In this meta-analysis of TKI monotherapy for advanced HCC, antitumor activity was broadly comparable between baseline Child–Pugh B and A: pooled RRs showed no significant differences for ORR and DCR. For safety, CP-B tended to experience more grade ≥3 trAEs (CP-A vs. CP-B RR 0.79, 95% CI 0.63–1.00), a borderline finding that warrants caution. Heterogeneity was moderate for trAEs and ORR and substantial for DCR; exploratory subgrouping by response criteria (RECIST vs. mRECIST) and TKI class did not account for this variability. Overall, TKIs appear to have comparable efficacy in CP-B and CP-A, with a possible increase in severe toxicity among CP-B patients.

## 5 Discussion

This meta-analysis evaluated the safety and efficacy of tyrosine kinase inhibitors (TKIs) monotherapy in patients with advanced hepatocellular carcinoma (HCC), comparing outcomes between Child-Pugh class A (CPA) and Child-Pugh class B (CPB) patients. Our study provides an integrated analysis of multiple guideline-recommended TKIs, offering a comprehensive understanding of their use in patients with varying degrees of liver dysfunction.

### 5.1 Efficacy outcomes

The heterogeneity observed in efficacy outcomes could be partially explained by variations in follow-up time, as well as other factors like the geographical origin of the studies (Western vs. Asian), treatment protocols, and baseline liver function in the patient populations across studies.

Across included studies, antitumor activity was broadly comparable between baseline Child–Pugh B and Child–Pugh A. Pooled random-effects estimates showed no significant differences for ORR and DCR. The prediction intervals encompassed the null for both endpoints, indicating that true effects may vary across settings but generally do not support a consistent advantage for either group. Taken together, these findings suggest that, in the context of TKI monotherapy, baseline CP-B does not preclude radiologic benefit.

### 5.2 Safety profile

For grade ≥3 treatment-related adverse events (trAEs), CP-A had a lower risk than CP-B (CP-A vs. CP-B RR ≈ 0.79, 95% CI 0.63–1.00; I^2^ ≈ 62%), a borderline-significant difference. The 95% prediction interval (≈0.45–1.39) crossed unity, highlighting variability across clinical contexts and emphasizing the need for careful patient selection and close toxicity monitoring in CP-B. Overall, these data indicate comparable efficacy with a possible increase in severe toxicity among CP-B patients.

### 5.3 Comparison with previous studies

Our efficacy results are compatible with prior observational cohorts reporting broadly similar response rates between CP-A and CP-B under TKI monotherapy, despite more frequent dose modifications and earlier discontinuations in patients with poorer liver function. Safety signals observed here (higher risk of severe AEs in CP-B) also align with real-world sorafenib/lenvatinib registries, in which hepatic reserve and comorbidity burden influence tolerability. Differences across publications likely reflect case-mix (B7 vs. B8–B9), treatment line, and supportive-care practices, which are also consistent with the heterogeneity we observed.

### 5.4 Clinical implications

This study provides valuable insight into the clinical practice of using TKIs in CP-B patients, emphasizing the importance of individualized dosing, toxicity management, and shared decision-making in the treatment of advanced HCC. The findings suggest that, while TKIs show comparable efficacy between CP-A and CP-B patients, careful monitoring for adverse events in CP-B patients is critical. These results could help clinicians refine their treatment strategies and optimize patient outcomes by considering liver function and comorbidity burden.

In advanced HCC patients ineligible for curative therapy, these findings support consideration of TKIs in selected CP-B patients, provided that:

1. Dosing and reduction protocols are proactively individualized;
2. Early AE surveillance (e.g., hypertension, hand–foot reaction, fatigue) is implemented;
3. Decision-making integrates global liver reserve (e.g., ALBI grade, presence of ascites/encephalopathy), performance status, and patient preference.

Given comparable radiologic efficacy but potential for higher toxicity, shared decision-making and multidisciplinary management are essential, and early switch or dose adjustment should be anticipated if tolerability is limited.

This meta-analysis presents several limitations, including potential biases from the observational nature of included studies. Variability in outcome definitions and AE grading systems (e.g., different CTCAE versions) also contribute to heterogeneity. Further studies with more uniform reporting standards and individual patient-level data are needed to confirm these findings.

### 5.5 Future directions

Prospective registries and pragmatic trials enriching CP-B—and stratifying B7 vs. B8–B9 or ALBI grade—are needed to refine patient selection and dosing. Dose-optimization and toxicity-mitigation strategies should be tested prospectively. Harmonized imaging and AE reporting (RECIST/mRECIST alignment; standardized CTCAE thresholds) would reduce between-study variability. Individual-patient-data meta-analyses could clarify the roles of performance status, viral status, portal hypertension, and dose intensity. Finally, comparative work against modern ICI-based regimens in CP-B and combination sequencing strategies will be important to define best practice.

### 5.6 Conclusion

In advanced HCC treated with TKI monotherapy, radiologic efficacy (ORR, DCR) appears broadly comparable between CP-B and CP-A, whereas severe toxicity may be more frequent in CP-B. These findings support cautious, individualized use of TKIs in selected CP-B patients, with proactive toxicity management. Given the heterogeneity and observational nature of much of the evidence, prospective, stratified research is required to optimize treatment for this vulnerable population.
