# Supplementary figures and images for: Efficacy and safety of tyrosine kinase inhibitors in advanced hepatocellular carcinoma patients with Child-Pugh A and B cirrhosis: a meta-analysis
Source: Front Pharmacol. 2026 Mar 11;17:1690890. doi: 10.3389/fphar.2026.1690890 (PMC13013448; doi:10.3389/fphar.2026.1690890)

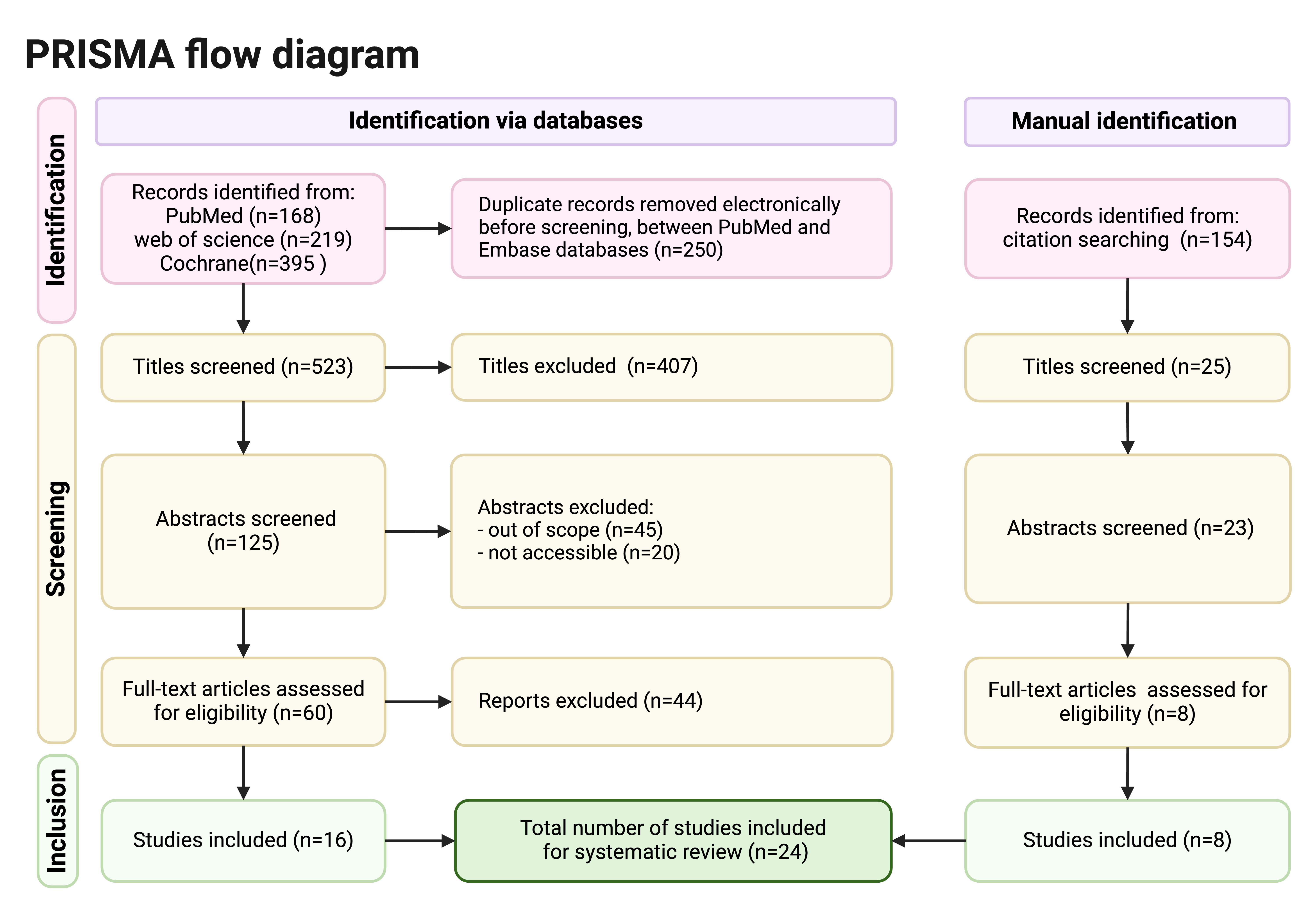

Supplement: Supplementary file 1 [file Image1.jpeg]
